# Mice transgenic for equine cyclin T1 and ELR1 are susceptible to equine infectious anemia virus infection

**DOI:** 10.1186/s12977-015-0163-7

**Published:** 2015-04-28

**Authors:** Cheng Du, Jian Ma, Qiang Liu, Yun-Fei Li, Xi-Jun He, Yue-Zhi Lin, Xue-Feng Wang, Qing-Wen Meng, Xiaojun Wang, Jian-Hua Zhou

**Affiliations:** State Key Laboratory of Veterinary Biotechnology, Harbin Veterinary Research Institute, Chinese Academy of Agricultural Sciences, Harbin, 150001 China; Department of Preventive Veterinary Medicine, College of Veterinary Medicine, Northeast Agricultural University, Harbin, 150001 China

**Keywords:** EIAV, Transgenic mouse, ELR1, CyclinT1, Infection

## Abstract

**Background:**

As a member of the tumor necrosis factor receptor (TNFR) protein superfamily, equine lentivirus receptor 1 (ELR1) has been shown to be expressed in various equine cells that are permissive for equine infectious anemia virus (EIAV) replication. The EIAV Tat protein (eTat) activates transcription initiated at the viral long terminal repeat (LTR) promoter through a unique mechanism that requires the recruitment of the equine cyclin T1 (eCT1) cofactor into the viral TAR RNA target element. *In vitro* studies have demonstrated that mouse fibroblast cell lines (e.g., NIH 3T3 cells) that express the EIAV receptor ELR1 and eCT1 support the productive replication of EIAV. Therefore, we constructed transgenic eCT1- and ELR1-expressing mice to examine whether they support *in vivo* EIAV replication.

**Findings:**

For the first time, we constructed mice transgenic for ELR1 and eCT1. Real-time reverse transcription polymerase chain reaction (RT-PCR) and Western blot analysis confirmed that ELR1 and eCT1 were expressed in the transgenic mouse tissues, particularly in the intestines, spleen and lymph nodes. Consistent with the results of EIAV infection in NIH 3T3 cells expressing ELR1 and eCT1, mouse embryonic fibroblasts (MEFs) from the transgenic mice could support EIAV replication. More importantly, this virus could infect and replicate in mouse blood monocyte-derived macrophages (mMDMs). Macrophages are the principle target cell of EIAV in its natural hosts. Furthermore, after the transgenic mice were inoculated with EIAV, the virus could be detected not only in the plasma of the circulating blood but also in multiple organs, among which, the spleen and lymph nodes were the predominant sites of EIAV replication. Finally, we found that consistent with high viral replication levels, the relevant pathological changes occurred in the spleen and lymph nodes.

**Conclusions:**

Our results show that mice transgenic for ELR1 and eCT1 are susceptible to EIAV infection and replication. Further, EIAV infection can cause lesions on the spleen and lymph nodes, similar to those frequently observed in horses, the natural hosts. Therefore, ELR1 and eCT1 are essential *in vivo* for EIAV invasion and replication.

**Electronic supplementary material:**

The online version of this article (doi:10.1186/s12977-015-0163-7) contains supplementary material, which is available to authorized users.

## Findings

Equine infectious anemia virus (EIAV) is the etiological agent for equine infectious anemia (EIA), a disease affecting equidae in most parts of the world. The infected animals, mainly horses and ponies, exhibit typical viremia, which is accompanied by fever, anemia, thrombocytopenia, edema, and weight loss. The virus maintains a certain level of stable replication in the peripheral blood and tissues, which are enriched with macrophages [1-3]. EIA is of considerable importance to the equine industry, and it is one of only 13 required reportable equine-specific diseases listed by the Office International Des Epizooties (OIE), the world organization for animal health [4]. Moreover, EIAV is a lentivirus that shares considerable homology with human immunodeficiency virus types 1 (HIV-1) and 2 (HIV-2). Therefore, progress in HIV-1 research can provide a reference for the study of EIAV [5]. An intact host response system in small animals that are susceptible to HIV-1 infection and replication will be helpful in studies of HIV-1 pathogenesis and host responses. There are several obstacles to establish a murine model of lentivirus infection and disease, particularly the activity of a series of host restrictions that impede critical steps in infection, gene expression, and virus assembly and budding [6-12].

Therefore, humanized mouse models, such as severe combined immunodeficient (SCID) mice implanted with human fetal thymus and liver [13] or Rag22/2cc 2/2 mice injected with human hematopoietic stem cells (hHSCs) [14], have been developed and used for HIV-1 investigations. However, the construction of these humanized mouse models is technically challenging, time-consuming and expensive; in addition, these models cannot take advantage of the wide array of available transgenic and gene-deleted mouse lines to apply genetic approaches to investigate HIV-1 transmission. Other methods have been used to overcome some of these defects, such as transgenic mice constructed to express human CD4, CCR5 and cyclin T1. These mice can support HIV-1 replication and are used for preliminary treatment assessments [15]. The combination of CD4 and the co-receptors CCR5 or CXCR4 can mediate the cell entry of HIV-1, and human cyclin T1 interacts with the viral Tat protein in a species-restricted manner to promote virus RNA transcription and processing [12]. These human-derived proteins are necessary for the HIV-1 infection of mouse cells.

ELR1, a member of the TNFR superfamily, has been identified as the sole receptor for EIAV [16]. The positive transcription elongation factor b (P-TEFb), which is composed of cyclin T and cyclin-dependent kinases 9 (CDK9), can initiate the transition from abortive to productive elongation. Cyclin T has three forms (T1, T2a and T2b); cyclin T1 is the major form. The histidine-rich domain of cyclinT1 is critical for P-TEFb to recognize and bind to RNA polymerase II [12]. Moreover, the HIV-1 Tat protein or EIAV activates transcription initiated at the viral long terminal repeat (LTR) promoter region through a species-restricted mechanism, which requires the recruitment of human or equine cyclinT1 (eCT1) as a cofactor to the viral TAR RNA target element [17].

NIH 3T3 cells (a cell line derived from mouse embryonic fibroblasts) that express the EIAV receptor ELR1 and eCT1 have been generated previously; these cells could support the productive replication of EIAV and the production of infectious virions at levels similar to those detected in the permissive equine dermal cell line (ED) [18]. However, there have been no further reports on the generation of transgenic mice expressing both ELR1 and eCT1. It was unclear whether the introduction of these equine proteins may result in a permissive environment for EIAV infection and replication similar to that observed in *in vitro* studies. To investigate this phenomenon, we generated transgenic mice carrying the ELR1 and eCT1 genes by microinjecting ELR1 and eCT1 recombinant plasmids together into fertilized oocytes from B6D2F1 (C57BL/6 × DBA/2F1) mice. This procedure was performed at the Liaoning Key Facility of Transgenic Laboratory Animals, China Medical University (Beier Road No.92, Heping District, Shenyang, Liaoning Province, China).

**Figure 1:**
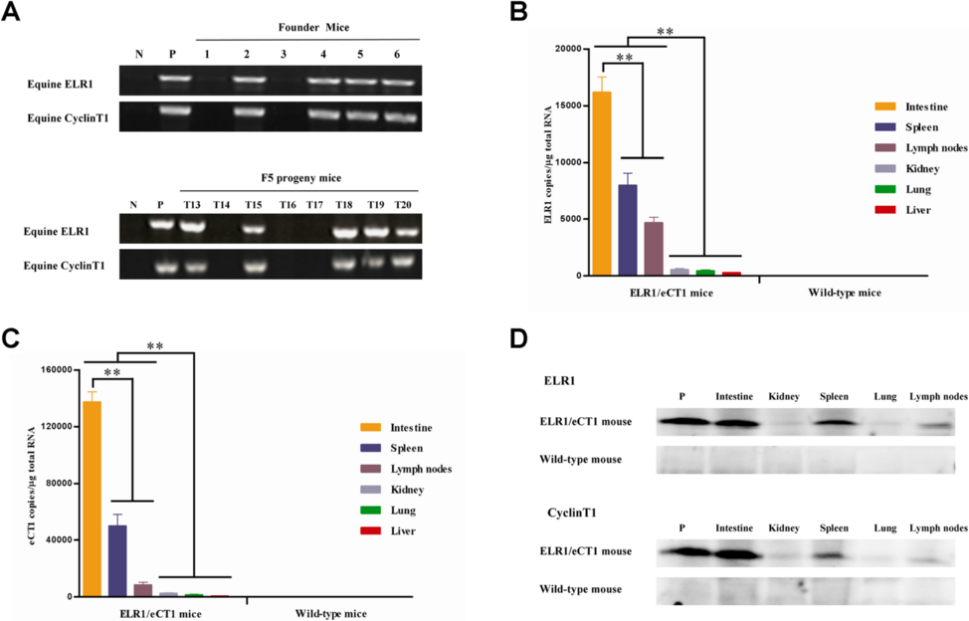
Evaluation of the transgene expression in ELR1/eCT1 mice. **(A)** The transmission of eCT1 and ELR1 in six transgenic founder mice (numbers 1–6) and eight F5 progeny mice (T13-T20) was determined by PCR. DNA was extracted from the tails of the indicated mice, and the integration of the ELR1 and eCT1 genes was detected by PCR with primer pairs specific for these two equine genes. N: negative control using wild-type mouse tail DNA; P: positive control using the ELR1 or eCT1 recombinant plasmid. **(B)** and **(C)** The ELR1 and eCT1 RNA levels in six organs (intestine, spleen, lymph nodes, kidney, lung and liver) from ELR1/eCT1 mice and wild-type mice (eight mice/group) were quantified by real-time RT-PCR. Statistical analyses were performed using SAS version 9.0 (SAS Institute Inc., Cary, NC). Significant differences between the organs in the groups of ELR1/eCT1 mice were determined using Student’s *t* test. *, *P* < 0.05; **, *P* < 0.01. **(D)** The expression of the ELR1 and eCT1 transgenes in the organs of the F5 progeny mice was detected by Western blot analysis. The lysates from the organs of the F5 progeny mice and wild-type mice were prepared, and equal amounts of protein (50 μg protein/sample) were used for the analysis. A specific monoclonal antibody was used to detect ELR1, and a rabbit antiserum was used to detect eCT1. P: positive control using lysates from 293T cells transfected with either the ELR1 or eCT1 recombinant plasmid.

The integration of exogenous ELR1 and eCT1 was identified in the genomes of four of six founder mice (from tail DNA samples) using polymerase chain reaction (PCR) (Figure 1A), with the equine-specific internal primer sets ELR1IN-F/ELR1IN-R and eCT1IN-F/eCT1IN-R, as shown in Additional file 1: Table S1. To obtain a uniform genetic background, these four ELR1- and eCT1-positive founder mice were backcrossed five times with C57BL/6 wild-type mice, which generated fifth-generation positive transgenic mice (Figure 1A).

To detect the transcription and protein expression levels of ELR1 and eCT1 in different organs of the transgenic mice, real-time reverse transcription PCR (RT-PCR) was performed using the primer sets ELR1QP-F/ELR1QP-R and eCT1QP-F/eCT1QP-R, as shown in Additional file 1: Table S1. The results showed that the two transgenes were not detected in wild-type mice (n = 8). In the transgenic mice (n = 8), the intestine was the organ in which the highest levels of ELR1 and eCT1 mRNAs were detected. In addition, remarkably higher ELR1 and eCT1 mRNA levels were expressed in the spleen and lymph nodes than in the kidney, lung or liver, with the lowest transgenes’ mRNA level close to the background level (Figure 1B and C). The expression of these two exogenous proteins was examined by Western blot analysis, with a monoclonal anti-ELR1 antibody or rabbit anti-eCT1 antibody as the first antibody, followed by incubation with an Alexa Fluor 800-labeled goat anti-mouse IgG or goat anti-rabbit IgG secondary antibody (Odyssey, USA). As shown in Figure 1D, the protein expression was consistent with the transcription level in the organs of the transgenic mice and wild-type mice; the strongest expression of both ELR1 and eCT1 occurred in the intestine. These data indicate that the transgenic mice were successfully constructed and that the ELR1 and eCT1 genes were stably expressed.

**Figure 2:**
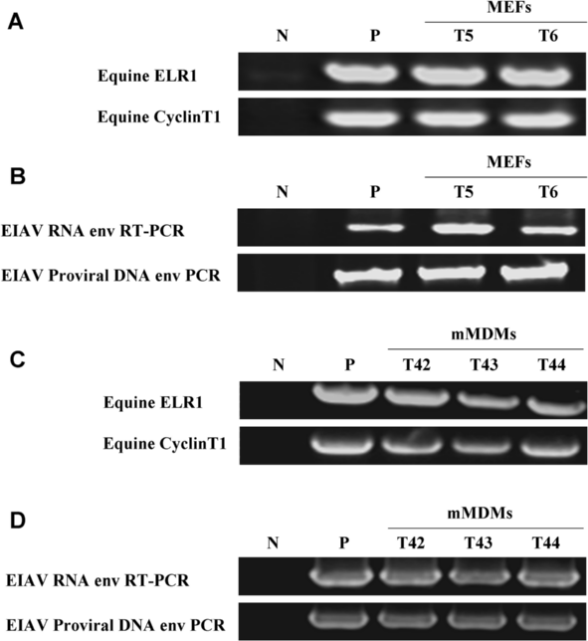
*In vitro* infection of mouse embryonic fibroblasts (MEFs) and mouse blood monocyte-derived macrophages (mMDMs) from ELR1/eCT1 transgenic mice with EIAV. **(A)** and **(C)** MEFs and mMDMs were prepared from the embryos and blood of ELR1/eCT1 mice and wild-type mice. Cellular DNA was extracted and assessed for the existence of ELR1 and eCT1 using PCR. N: negative control using MEFs and mMDMs from wild-type mice; P: positive control using equine monocyte-derived macrophages (eMDMs). **(B)** and **(D)** MEFs and mMDMs prepared from ELR1/eCT1 mice and wild-type mice, as well as eMDMs, were infected with 10^3^ TCID50 EIAV_DLV34_. The cells were washed three times and then cultivated for 72 h. The viral RNA was extracted from the culture supernatant and amplified by RT-PCR using EIAV-*env* specific primers. In addition, total DNA was prepared from the lysates of the same sets of 72 h-cultivated cells to detect the proviral genomes of EIAV using PCR with the same primers. N: negative control using MEFs and mMDMs from wild-type mice; P: positive control using eMDMs.

To determine the susceptibility of the cells from these transgenic mice to EIAV infection *in vitro*, mouse embryonic fibroblasts (MEFs) and blood monocyte-derived macrophages (mMDMs) isolated from the ELR1- and eCT1-expressing transgenic mice and wild-type mice were prepared, as previously described [19,20], and the cells were then incubated with 10^3^ TCID50 of an EIAV pathogenic strain EIAV_DLV34_ (a donkey MDM-adapted virulent strain that was obtained from 34 passages that retained pathogenicity to horses), after detecting the ELR1 and eCT1 genes by PCR (Figure 2A and C). The cells were washed three times 24 hours post-inoculation to remove free virions, and the culture supernatants were evaluated for the presence of the EIAV envelope gene (*env*) using PCR 72 hours later, as previously described [21]. Productive EIAV infections were indicated by the presence of the *env* fragment in the culture supernatant of both the MEF and mMDM cultures from the transgenic mice, but not the wild-type mice (Figure 2B and D). Moreover, PCR was also performed for the EIAV proviral *env* DNA. Infections by EIAV_DLV34_ were further confirmed by the presence of *env* DNA, which indicates the integration of EIAV in the target cells (Figure 2B and D). These results are consistent with previous studies showing that NIH 3T3 cells expressing ELR1 and eCT1 supported the productive replication of EIAV [18], but these findings also prove that EIAV can replicate in mMDMs from the transgenic mice. Macrophages are the principle target cells for EIAV infection in equids.

**Figure 3:**
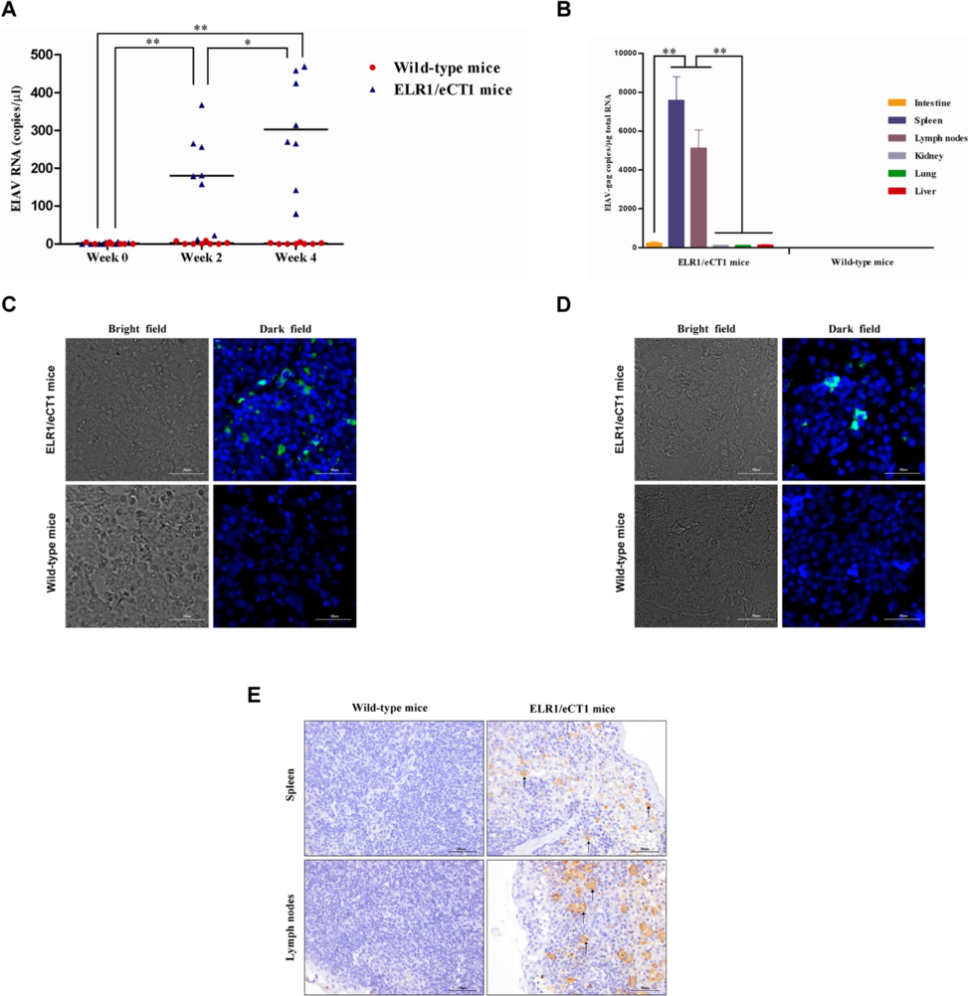
*In vivo* EIAV infection of ELR1/eCT1 mice with EIAV. **(A)** The plasma viral loads of EIAV in ELR1/eCT1 and wild-type mice (n = 8 for each group) were measured using quantitative real-time RT-PCR for the *gag* gene at 0, 2 and 4 weeks after intraperitoneal injection with 10^4^ TCID50 EIAV_DLV34_. *, *P* < 0.05; **, *P* < 0.01, using Students *t* test, between the ELR1/eCT1 mice groups at 0, 2 and 4 weeks after intraperitoneal injection. **(B)** Copies of the EIAV genomic RNA in six organs (intestine, spleen, lymph nodes, kidney, lung and liver) from ELR1/eCT1 mice and wild-type mice (n = 8 for each group) were measured after intraperitoneal injection with EIAV_DLV34_ for four weeks. *, *P* < 0.05; **, *P* < 0.01, using Student’s *t* test. **(C)** and **(D)** RNA-RNA fluorescence *in situ* hybridization (FISH) of spleen and lymph node sections from ELR1/eCT1 and wild-type mice was performed after intraperitoneal injection with EIAV_DLV34_ for four weeks. The existence of EIAV was detected through the hybridization of viral genomic RNA with EIAV-specific RNA probes that were labeled with digoxigenin (DIG) and then stained with anti-DIG-fluorescein, Fab fragments (green fluorescence). The cell nuclei were stained with 4′,6-diamidino-2-phenylindole (DAPI) (blue fluorescence). **(E)** Immunohistochemistry was performed to detect EIAV proteins in the spleen and lymph nodes from ELR1/eCT1 and wild-type mice after intraperitoneal injection with EIAV_DLV34_ for four weeks. The tissue sections were stained with a monoclonal antibody recognizing the EIAV p26 antigen and a horseradish peroxidase (HRP) conjugated anti-mouse IgG. The cells infected with EIAV are shown as brown (typical cells are indicated by arrows).

The *in vivo* infectivity and proliferation of EIAV in ELR1/eCT1 transgenic mice were further evaluated by inoculating (i.e., by intraperitoneal injection) ELR1/eCT1 mice (n = 8) and wild-type mice (n = 8) with 10^4^ TCID50 EIAV_DLV34_. Subsequently, using real-time RT-PCR, the copy number of the virus was detected in the plasma of peripheral blood obtained at week 0 (W0, immediately after the injection), as well as at weeks 2 (W2) and 4 (W4) (post-injection using the primers EIAV-gag-F and EIAV-gag-R), as shown in Additional file 1: Table S1. As demonstrated in Figure 3A, the development of productive infection was indicated by the absence of detectable plasma viremia in all wild-type mice after infection, while the plasma viremia levels in the ELR1/eCT1 mice increased extremely significantly from 0 to 2 weeks and also increased significantly from 2 to 4 weeks after the inoculation.

In addition, the virus distribution in the organs of the infected mice was investigated. Different from the relative expression levels of ELR1 and eCT1 mRNA and protein, which were most highly expressed in the intestine, the EIAV genomic RNA was detected in two secondary lymphoid organs, the spleen and lymph nodes, at significantly high copy numbers (7582 ± 1218 and 5117 ± 933 copies/μg RNA, respectively); however, the viral RNA was only slightly above detectable levels (approximately 100 copies/μg) in the intestine, kidney, lung and liver (Figure 3B).

Furthermore, to provide direct evidence of EIAV infection and proliferation in ELR1/eCT1 mice, EIAV RNA and protein in the infected spleen and lymph nodes were examined using RNA-RNA fluorescence *in situ* hybridization (FISH) and immunohistochemistry (IHC), respectively. The EIAV-specific RNA probes used in FISH are shown in Additional file 1: Table S1. A monoclonal anti-EIAV-p26 antibody was used for IHC. The ELR1/eCT1 mice and control mice were sacrificed at W4 of the EIAV infection. The organs were examined for the presence of EIAV. The FISH results demonstrated that fluorescent hybridization signals were detected in the spleen and lymph nodes of the ELR1/eCT1 mice (Figure 3C and D). Consistently, the IHC analysis also clearly showed that numerous cells were stained positive for the EIAV capsid protein in the spleen and lymph nodes of the ELR1/eCT1 mice. Neither FISH- nor IHC-positive cells were observed in the wild-type control mice (Figure 3E).

**Figure 4:**
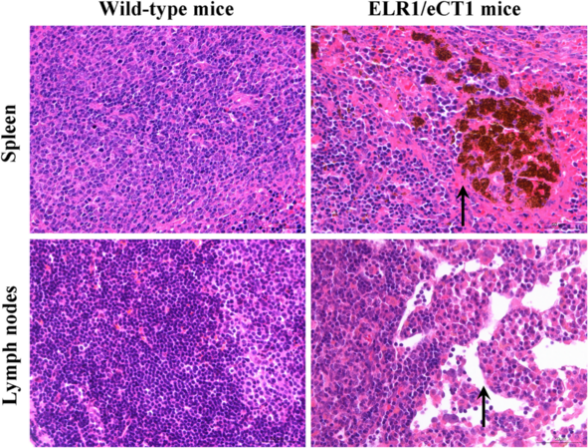
Pathologic changes in the spleen and lymph nodes of the transgenic mice. Tissue sections from the intestines, spleens, lymph nodes, kidneys, lungs and livers of ELR1/eCT1 and wild-type mice were prepared after intraperitoneal injections with EIAV_DLV34_ for four weeks. The pathologic changes of organs stained with hematoxylin-eosin were analyzed by optical microscopy. Large areas of focal hemosiderin deposits were observed in the spleen (arrows), but not in the other organs of ELR1/eCT1 mice. In addition, no such areas were observed in wild-type mice. The medullary lymphocytes decreased significantly in the lymph nodes (arrows), but not in the other organs of ELR1/eCT1 mice. No such decrease was observed in wild-type mice.

To investigate whether the proliferation of EIAV exerts a pathogenic effect on the involved organs, morphology changes in the tissues were examined by hematoxylin-eosin staining under optical microscopy. Significantly decreased numbers of lymphocytes were observed in the outer cortex or inner medulla region of the lymph nodes of ELR1/eCT1 mice compared with the organs from wild-type control mice. Furthermore, mononuclear phagocyte-like cells, with an extensive accumulation of deposited hemosiderin, were found clustering at many sites in the spleens of the transgenic mice (Figure 4).

Previous studies of organs from horses acutely infected with EIAV demonstrated that the high levels of circulating viral load observed during acute EIA were associated with high levels of virus replication in macrophage-rich organs, including the spleen, lymph nodes, liver and kidney [2,22,23]. Our results showed that EIAV replicated to high titers in ELR1/eCT1 mice spleens and lymph nodes, but not in other organs, such as the liver and kidney (Figure 3B). We speculate that this result may be due to the low transcription levels of ELR1 and eCT1 in these organs (Figures 1B and C). In addition, the intestinal immune system is severely affected by commensal microorganisms from the intestinal tract, which can restrict viral replication [24]. The mouse intestinal microflora differ from equine intestinal microflora; in addition, there may be other unknown restriction effects against EIAV in the mouse intestine. Therefore, the intestinal immune system may restrict EIAV replication, leading to ineffective viral replication even with the higher expression levels of ELR1 and eCT1 in the intestine (Figures 1B, C, D and 3B).

After infection by EIAV, *in vivo* circulating virus-antibody complexes can bind to erythrocytes through the Fc receptor or the complement receptor; next, complement is activated through the classical pathway, resulting in the phagocytosis of the involved erythrocytes by macrophages and neutrophils and leading to hemolysis. Large amounts of hemosiderin are regularly observed in the spleens of acutely EIAV-infected horses [25]. In the present study, after infection by EIAV, large areas of focal hemosiderin deposits were detected in the spleens of ELR1/eCT1 mice but were absent in wild-type mice infected with EIAV (Figure 4). Moreover, in ELR1/eCT1 mouse lymph nodes (but not in wild-type mouse lymph nodes), the medullary lymphocyte numbers were significantly decreased (Figure 4). This result is consistent with those of previous studies of the tissue lesions of EIAV-infected horses [26]. Together, these studies indicate that EIAV infection of ELR1/eCT1 mice can result in pathological organ changes similar to those observed in infected horses; the results show that EIAV can replicate in mMDMs from ELR1/eCT1 mice, suggesting the potential application of ELR1/eCT1 mice as a small laboratory animal model for EIA.

In conclusion, we constructed for the first time a novel fully transgenic mouse (ELR1/eCT1 mice) that supports the *in vivo* replication of EIAV, and we showed that ELR1 and eCT1 play a vital role in EIAV replication and production *in vivo*. Because it is more expensive to use horses in animal experiments and horse-related antibodies and reagents are not easily obtained, ELR1/eCT1 mice may provide an alternative *in vivo* infection model that is highly reproducible, inexpensive and widely available for evaluating the effectiveness of candidate EIAV vaccines and for studying the pathobiology of EIAV infection.

### Ethics statement

Animal experiments were approved by Animal Ethics Committee of Harbin Veterinary Research Institute of the Chinese Academy of Agricultural Sciences (CAAS) and performed in accordance with animal ethics guidelines and approved protocols. The Animal Ethics Committee approval number was SYXK (Hei) 2011022.

## Additional file

Additional file 1: Table S1. The following oligonucleotides were used for the PCR, real-time RT-PCR and RNA-FISH assays. The name refers to the DNA or RNA target.
