# Supplementary material for: Mice transgenic for equine cyclin T1 and ELR1 are susceptible to equine infectious anemia virus infection
Source: Retrovirology. 2015 Apr 28;12:36. doi: 10.1186/s12977-015-0163-7 (PMC4422544; doi:10.1186/s12977-015-0163-7)
Supplement: Additional file 1: Table S1. — The following oligonucleotides were used for the PCR, real-time RT-PCR and RNA-FISH assays. The name refers to the DNA or RNA target. [file 12977_2015_163_MOESM1_ESM.doc]

**Supplemental Table 1:** The following oligonucleotides were used for the PCR, real-time

| *Name* | *Sequence(5’-->3’)* |
| --- | --- |
| ELR1IN-F | 5’-GGCCACTTCTGCGTTAGCGAG-3’ |
| ELR1IN-R | 5’-TGGGTAGGCCTGGCAGCTCTC-3’ |
| eCT1IN-F | 5’-GCAAGTGGTCCAACTGGGAG-3’ |
| eCT1IN-R | 5’-GAACCATCCTGTTGCAAGGAATG-3’ |
| ELR1QP-F | 5’-TCAGTGGACACGTCCTGGTG-3’ |
| ELR1QP-R | 5’-TCGGTCAGCATGTTGCTCCCT-3’ |
| eCT1QP-F | 5’-ACAGGATGGTTCGAATGCATTTAT-3’ |
| eCT1QP-R | 5’-AGTGTCACTGAAAGAATACCGTGC-3’ |
| EIAV-gag-F | 5’- CGATGCCAAATCCTCCATTAG -3′ |
| EIAV-gag-R | 5’- CTGATCAAAAGCAGGTTCCATCT -3′ |
| EIAV-specific probe A | 5’- CCCCTCTGTCATTGCTTCTATTATCCACTGTCT -3’ |
| EIAV-specific probe B | 5’- CCCTAATGAACCTTGCTGTCATGGGAATAGGC -3’ |
| EIAV-specific probe C | 5’- GTGTTTTGTTCATTGATTCCTTCTGCACAGGGA -3’ |

RT-PCR and RNA-FISH assays.The name refers to the DNA or RNA target.
